# Examining ecosystem services and disservices through deliberative socio-cultural valuation

**DOI:** 10.1007/s43545-022-00511-8

**Published:** 2022-09-22

**Authors:** D. S. Baltazar, J. Labadz, R. Smith, A. Telford, M. Di Bonito

**Affiliations:** 1 School of Animal, Rural, and Environmental Sciences, Nottingham Trent University, Brackenhurst Ln, Southwell, Nottingham, NG25 0QF UK; 2 Nottingham Business School, Nottingham Trent University, 50 Shakespeare Street, Nottingham, NG1 4FQ UK; 3 Faculty of Humanities, University of Amsterdam, Kloveniersburgwal 48, 1012 CX Amsterdam, The Netherlands

**Keywords:** Deliberative socio-cultural valuation, Ecosystem services and disservices, Urban parks

## Abstract

The deliberative socio-cultural valuation of ecosystem services (ES) and disservices (EDS) is an understudied area of ES and EDS research. Participatory methods have been applied to ES and EDS valuation, but little is known on how these approaches could reveal and form shared values and impact decision-making. This paper presents the deliberative socio-cultural valuation of the Jose Rizal Plaza in Calamba City, The Philippines. The study aimed to assess how stakeholders value the ES and EDS of the park and examine how these values change in different situations. Online focus groups were carried out, and in each, the participants were asked to distribute importance and concern points to the various park ES and EDS, respectively. The valuation exercise was performed six times, changing the source and constituency of the valuation, and introducing discussions. Results confirm significant differences in the values assigned to several ES and EDS across the valuation exercises. Varying the sources and constituencies proved useful in revealing the participants’ shared assigned values. The participants share a high appreciation for enjoyment and spending free time, sports and physical fitness, relaxation and mental recreation, social relationships, and local identity and cultural heritage. For EDS, they share a significant concern only for the risk of anti-social behaviour. This type of valuation could be further explored using other parks and cities to test if it will have consistent results. For the Jose Rizal Plaza, spaces for sports should be maintained and security should be improved.

## Introduction

There has been a growing interest in the socio-cultural valuation of ecosystem services (ES) and disservices (EDS) in recent years. This might have come from recognising that the two other types of ecosystem service valuation, economic and ecological, do not provide a holistic representation of the value of these benefits and disbenefits. This is especially true when the ecosystem of interest possesses more intangible and abstract ES and EDS. Economic valuation transforms the value of ES and EDS into monetary terms (Hodgson et al. 2012), while ecological valuation evaluates specific ecosystem attributes to determine their importance in maintaining the ecosystem’s overall health and functions (Small et al. 2017). In contrast, socio-cultural valuation explores how people assign values to the different ES and EDS, considering their socio-economic backgrounds, held values, and preferences (Iniesta-Arandia et al. 2014; Ruiz-Frau et al. 2018).

In conducting socio-cultural valuation, it is fundamental to distinguish between “held” and “assigned” values (Brown 1984). Held values are sets of qualities or end states (e.g., wisdom, happiness, freedom) or modes of conduct (e.g., generosity, courage, obedience).

that influence a person’s evaluative judgment. On the other hand, assigned values are the actual expression of the importance of an object relative to another (Brown 1984; Sánchez-Fernández and Iniesta-Bonillo 2007). They arise from a person’s set of held values and their relationship with the object being valued. They are also thought to be affected by the valuation context (Brown 1984; Kenter et al. 2016a, b, c). This context relates to the valuator’s physical and emotional states, external situation (e.g., financial situation, free time, environmental conditions) and aspirations for future changes in that situation, the constituency of valuation, and the overall process of elicitation (Brown 1984; Kenter et al. 2016a, b, c).

There are two common methods of eliciting assigned values—individual and through deliberations. Individual elicitation is accomplished through interviews and surveys, while deliberations can be undertaken through focus groups or workshops. There is a wealth of socio-cultural valuation studies that utilise individual elicitation of assigned values (see Langemeyer et al. 2015; Maestre-Andrés et al. 2016; Schmidt et al. 2017), while the use of deliberations or discussions remains an understudied area of socio-cultural valuation (Borsuk et al. 2019; Kobryn et al. 2018). Moreover, little is known on how these participatory approaches could reveal and form shared values and impact decision-making.

This paper presents a deliberative socio-cultural valuation of an urban park in Calamba City, the Philippines, using online focus groups. It attempts to illustrate how deliberations could impact assigned values to ES and EDS. In doing so, it hopes to encourage more cities to consider making stakeholder deliberation a constant practice in designing and maintaining urban parks. The two main objectives of the study are to a) assess how stakeholders value the park ES and EDS and b) examine how values change when the source and constituency of the valuation are modified and when communication is introduced. The paper is structured in four sections – Introduction, Methods, Results and Discussion, and Conclusion.

## Methods

### Recruitment of participants

The focus group participants were recruited through an online valuation survey conducted in the earlier part of the study and through social media posts. The focus groups were carried out online through Zoom Videoconferencing Software (Zoom Video Communications Inc, 2016). Eight focus groups with three participants each were conducted from July to August 2020 and were all facilitated by the author.

### Focus group structure and procedure

In each focus group, the participants were first sent a link to an online consent form and entry questionnaire. The entry questionnaire consisted of three sections—(1) park use; (2) Social value orientation (Murphy ans Ackermann 2013); and (3) socio-economic characteristics. The participants were then asked to listen to a brief presentation about the concept of ES and EDS and the Jose Rizal Plaza and its ES and EDS. The list of ES and EDS was from key informant interviews conducted during the initial stages of the research. Representatives of different stakeholder groups identified with the help of the city office were asked what benefits and disbenefits they think the park has. The stakeholder groups were the residents living in the villages closest to, near, and far from the park (based on public transportation), city office employees, and college students.

The participants of each focus group were then asked to freely distribute 100 hypothetical “importance points” to the various park ES and 100 hypothetical “concern points” to the park EDS (Johnson et al. 2019; Schmidt et al. 2016). Importance and preference points were used instead of a currency because socio-cultural valuation does not focus on monetary values (Iniesta-Arandia et al. 2014). Moreover, the points were limited to 100 to emphasise the idea of trade-offs. This valuation exercise was performed six times—four times individually and two times as a group, in different situations (Table 1). The situations were based on the changes in the source and constituency of their valuation and their interaction among the participants. The interaction was introduced by asking the participants to distribute the points as a group and letting them discuss trade-offs and future generations. Participants were informed that trade-offs arise from the deliberate or unintended optimisation of a few ES, leading to the deterioration of other ES because of human management choices (Rodríguez et al. 2006).

**Table 1:** Value source and constituency of the valuation exercises performed by the focus group participants

Valuation	Value source	Value constituency
1	Individual	Self
2	Group	Individual
3	Individual	Group
4	Individual	Future generations
5	Group	Future generations
6	Individual	Future generations (after discussions)

The constituency of valuation is the subject to which the valuation is performed. According to Brown (1984), there are four value source and constituency combinations, namely, individual to self, individual to group, group to individual, and group to group. This study included only the first three since it is challenging to manage multiple groups in an online setting. Two additional value source and constituency combinations were added, individual to future generations and group to future generations, to assess how participants respond when asked to make choices on behalf of the future generations. Individual to future generation valuations were repeated after group deliberations to determine how discussions could affect the values assigned to ES and EDS. For the individual valuations, the participants were given links to valuation forms. For the group valuations, the participants were asked to voice out the ES and EDS that they think are important or concerning. They were then asked to cast votes for the ES and EDS that were put forward, after which the percentage of votes were computed to represent the importance and concern points for ES and EDS, respectively.

After the valuations, a debriefing session was carried out to ask the participants how they think the different situations affected how they distributed points among the park ES and EDS and what they learned from the focus group. The focus group was concluded by an exit questionnaire which asked the participants the concepts they learned through the focus group. The focus group guidelines, questionnaires, and valuation forms are in the Supplementary materials. All the questionnaires and valuation forms were made available online through the Qualtrics Core XM Survey Tool. The discussions were video recorded and transcribed, and the valuation data from Qualtrics was exported as an SPSS data set for analysis.

### Study area: The Jose Rizal Plaza in Calamba City, The Philippines

**Fig. 1:**
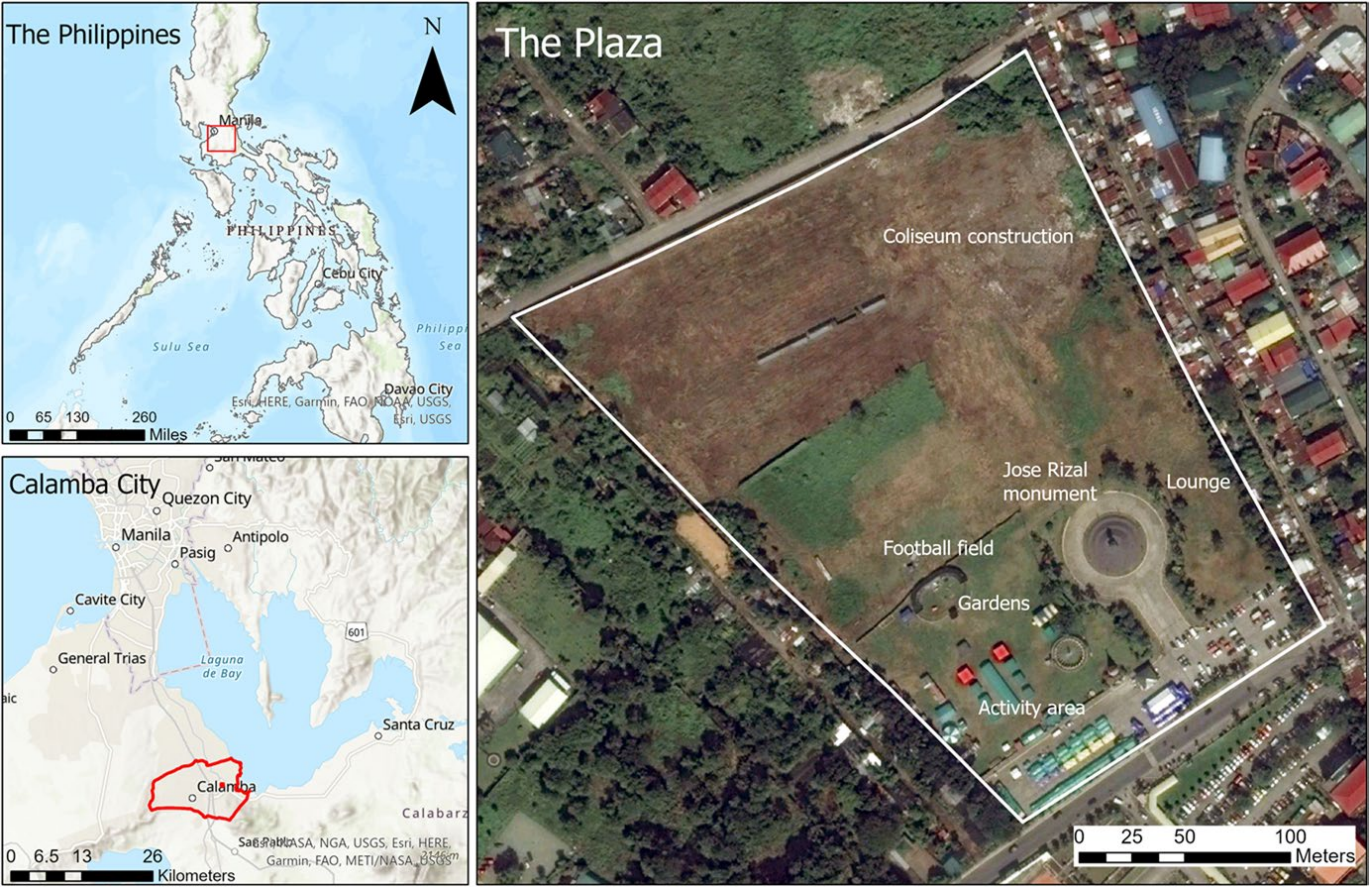
The Jose Rizal Plaza in Calamba City, The Philippines

The Jose Rizal Plaza is a park geographically located in north latitude 14° 11′ 47.76" and east longitude 121° 9′ 33.12" in the City of Calamba, province of Laguna, The Philippines. It has an area of seven hectares and has the following amenities: football field, gardens, lounge (which has not been opened yet to the public), and activity area (used for Zumba classes, jogging, and different events) (Fig. 1). Calamba City has an area of 144.80 km^2^ and is the second-largest city in the Laguna Province. It is about 45 km away from the Metro Manila Region and has a population of 454,486 as of 2015 (Calamba City 2017).

### Data analysis

Responses from the entry and exit questionnaires and the valuation forms were anonymised and then analysed using IBM SPSS Statistics for Windows, Version 26.0. Shapiro–Wilk’s tests (Shapiro and Wilk 1965) were applied to the various park ES and EDS valuation scores in the six valuation exercises. It was found that only a few follow a normal distribution (*p* > 0.05). For this reason, non-parametric tests were used to compare the distributions. The discussion transcripts were analysed using summative content analysis (Hsieh and Shannon 2005; Kondracki et al. 2002). Keywords from the participants’ comments were identified and coded according to the ES or EDS that they referred to and the specific questions during the debriefing session using Microsoft Word’s comment function. They were then extracted and collated into a spreadsheet, after which their general themes were identified. The datasets generated during and/or analysed during the current study are available from the corresponding author on reasonable request.

## Results and discussion

### Socio-economic characteristics

A little more than half of the participants (54.17%) found out about the focus groups from a friend or a relative, and the rest found it through the author, social media posts, and city and village office employees. Other socio-economic characteristics of the participants are listed in Table 2. The participants’ mean age is 28.25 (*SD* = 8.48), with the most common ages being 18, 30, and 32. The youngest participant is 18, and the oldest is 56. More than half of the participants were female (54.2%), while 45.8% were males. Categorising the participants according to the stakeholder groups that were identified in the earlier stages of the research, most of them were residents of villages near the park (within a 4-km radius from the park) (41.67%) and college students (37.5%). Few were city office employees (8.33%) and residents of villages closest (“Halang” and “Real” village) (8.33%) and far from the park (outside a 4-km radius from the park) (4.17%).

**Table 2:** Socio-economic characteristics of the focus group participants

Socio-economic characteristics	Categories	Statistics (*N* = 24)
Age		*M* = 28.25; *SD* = 8.48
Gender	Female	13 (54.2%)
	Male	11 (45.8%)
Marital status	Single	17 (70.8%)
	Married	7 (29.2%)
House ownership	Owned	10 (41.7%)
	Rented	8 (33.3%)
	Shared	3 (12.5%)
	Mortgaged	2 (8.3%)
	Others (not specified)	1 (4.2%)
Educational attainment	Complete college	10 (41.7%)
	Graduate school	9 (37.5%)
	Complete high school	2 (8.3%)
	Incomplete college	2 (8.3%)
	Incomplete high school	1 (4.2%)
Migrant	No	18 (75%)
	Yes	6 (25%)

Most focus group participants come from villages near (within a 4-km radius) the park. It can be observed from the demographic data that the participants were relatively young and had high educational attainment. This was deemed to be the consequence of promoting and administering the focus groups online because of the Coronavirus pandemic. Younger people and those who are well-educated can be assumed to have more knowledge about mobile phones, computers, and the internet, which became a requirement in participating in the online focus groups. This can be considered as a limitation of the study as those less educated and older members of the community were not represented in the study. Future studies should look into recruiting more participants and conducting the focus groups face to face.

### Deliberative valuation of ecosystem services (ES) and disservices (EDS)

#### Individual to self

Table 3 lists the park ES that the participants valued the most and the least when asked to think only about their own interests. The reasons given by the participants in assigning higher points to ecotourism come from their opinions and personal experiences. For example, some participants mentioned that they believe ecotourism could lead to other park ES like revenue for the city and additional income for the residents:

Ecotourism; because it can create a domino effect. If you have ecotourism, you can promote relaxation… More people will visit the park, and the city office will benefit from the tax [coming from vendors].

**Table 3:** List of ES that the participants valued most and the least in the different valuation exercises: 1—Individual to self, 2—Group to individual, 3—Individual to group, 4—Individual to future generations, 5—Group to future generations, and 6—Individual to future generations (after discussions)

Valuation	ES valued most	ES valued least*
1	Ecotourism (*M* = 16.42, *SD* = 9.69, *Mdn* = 16.5); sports and physical fitness (*M* = 15.79, *SD* = 12.37, *Mdn* = 10); enjoyment (*M* = 13.04, *SD* = 12.02, *Mdn* = 10); relaxation and mental recreation (*M* = 12.67, *SD* = 10.80, *Mdn* = 10)	Improving residents’ non-economic quality of life (M = 0.75, SD = 2.17); city revenue (M = 1.33, SD = 2.75); park’s use as a parking space (M = 1.79, SD = 4.46); revenue for locals (M = 2.17, SD = 3.73)
2	Ecotourism (*M* = 19.37, *SD* = 8.44, *Mdn* = 20.71); enjoyment (*M* = 15.04, *SD* = 7.30, *Mdn* = 13.33); sports and physical fitness (*M* = 14.93, *SD* = 6.65, *Mdn* = 13.94); relaxation and mental recreation (*M* = 11.45, *SD* = 6.42, *Mdn* = 11.11)	Park’s use as a parking space (*M* = 0); improving the residents’ non-economic quality of life (*M* = 0.78, *SD* = 2.11); information for cognitive development (*M* = 0.78, *SD* = 2.11); stimulate residents’ interest to history and culture (*M* = 2.17, *SD* = 4.04)
3	Ecotourism (*M* = 22.04, *SD* = 13.47, *Mdn* = 21.5); sports and physical fitness (*M* = 15.08, *SD* = 12.42, *Mdn* = 12.5), relaxation and mental recreation (*M* = 13.79, *SD* = 14.53, *Mdn* = 10); enjoyment (*M* = 12.58, *SD* = 11.08, *Mdn* = 11)	Park’s use as a parking space (*M* = 0.29, *SD* = 0.75); improving the residents’ non-economic quality of life (*M* = 0.79, *SD* = 2.26); city revenue (*M* = 1.71, *SD* = 4.57); information for cognitive development (*M* = 1.79, *SD* = 5.43)
4	Sports and physical fitness (*M* = 17, *SD* = 16.68, *Mdn* = 11.5); ecotourism (*M* = 12.17, *SD* = 10.21, *Mdn* = 11.5); enjoyment (*M* = 11.71, *SD* = 13.13, *Mdn* = 10); relaxation and mental recreation (*M* = 9.29, *SD* = 10.69, *Mdn* = 8)	Park’s use as a parking space (*M* = 0.42, *SD* = 1.21); city revenue (*M* = 0.83, *SD* = 2.68); improving the residents’ non-economic quality of life (*M* = 0.92, *SD* = 2.19); information for cognitive development (*M* = 3.13, *SD* = 4.57)
5	Sports and physical fitness (*M* = 16.56, *SD* = 6.17, *Mdn* = 15); ecotourism (*M* = 13.71, *SD* = 9.40, *Mdn* = 16.67); revenue for locals (*M* = 9.67, *SD* = 3.51, *Mdn* = 9.09); enjoyment (*M* = 10.03, *SD* = 7.24, *Mdn* = 15)	Park’s use as a parking space (*M* = 0); improving the residents’ non-economic quality of life (*M* = 0.83, *SD* = 2.25); city revenue (*M* = 1.39, *SD* = 3.75); provide aesthetic information (*M* = 1.53, *SD* = 2.72)
6	Ecotourism (*M* = 15.88, *SD* = 12.89, *Mdn* = 15); sports and physical fitness (*M* = 14.25, *SD* = 13.99, *Mdn* = 13.5); enjoyment (*M* = 11.67, *SD* = 10.74, *Mdn* = 10); relaxation and mental recreation (*M* = 11.25, *SD* = 9.15, *Mdn* = 10)	Park’s use as a parking space (*M* = 0.38 *SD* = 1.24); improving the residents’ non-economic quality of life (*M* = 1.13, *SD* = 3.44); city revenue (*M* = 1.71, *SD* = 5.08); space for events (*M* = 2.67, *SD* = 5.60)

*All the least valued ES had a median of zero.

Some said that ecotourism could aid in the city’s promotion and income generation, while others thought it was important because it enables their relatives from other cities to visit the park. Participants’ reasons for assigning higher points to sports, enjoyment, and relaxation were based more on their personal experiences. Some value the ES related to sports and physical fitness because they are members of sports organisations that run their events in the park. Some jog, run, play sports, and attend Zumba lessons in the park. The park’s ability to serve as a place for enjoyment and relaxation was important to them because they come to the park to de-stress, meet with friends and family, and enjoy the scenery, especially during holidays. Participants did not mention specific reasons in assigning lower points to some of the ES, but one participant expressed disappointment that the park is being used as a parking space:

It [the park] should be serving the public… Unfortunately, now, you can only see it being used as a parking space, which defeats its purpose.

A Kendall’s W was run to determine if there was an agreement on how the focus groups assigned points to the different ES. It was determined that the focus groups did not agree on how they assigned points to the different ES, W = 0.686, p < 0.001.

Table 4 lists the park EDS that the participants were most concerned and least worried about. Participants reported personal encounters of anti-social behaviour in the park, like bullying, gang fights, littering, vandalising, and crimes, that they think were primarily caused by the park’s poor security and allowing late-night gatherings in the area. They also expressed their concerns about the expensive maintenance of the park. Some fear that there is corruption in the city office, while others say that this could have been caused by not consulting the public about the facilities that they would like to have in the park:

Expensive construction and maintenance because the city spend a huge amount there.. and the corruption is always there…I know that because I grew up with some politicians.

When the coliseum was built, I and many people I know became worried because the city office spent a lot for it, but it made space [in the park] seem smaller. We thought that they could have just improved the park and not spent a lot for it [coliseum], because people were not consulted. Nobody consulted the people of Calamba if they really wanted this, only those ‘decision makers’ in the government decided that.

**Table 4:** List of EDS that the participants were most and least concerned about in the different valuation exercises: 1—Individual to self, 2—Group to individual, 3—Individual to group, 4—Individual to future generations, 5—Group to future generations, and 6—Individual to future generations (after discussions)

Valuation	EDS most worried about	EDS least worried about
1	Anti-social behaviour (*M* = 25.71, *SD* = 27.30, *Mdn* = 20); expensive maintenance (*M* = 20.92, *SD* = 22.25, *Mdn* = 18.5)	Conflict among users (*M* = 6.08, *SD* = 8.62, *Mdn* = 1); thought of the land being wasted (*M* = 7.92, *SD* = 14.58, *Mdn* = 3.5)
2	Anti-social behaviour (*M* = 23.16, *SD* = 8.30, *Mdn* = 20.71); expensive maintenance (*M* = 22.43, *SD* = 7.94, *Mdn* = 22.72)	Thought of the land being wasted (*M* = 0.89, *SD* = 2.41, *Mdn* = 0); exposure to air pollution (*M* = 5.8, *SD* = 6.61, *Mdn* = 3.57)
3	Traffic (*M* = 25.33, *SD* = 24.71, *Mdn* = 20); anti-social behaviour (*M* = 24.25, *SD* = 24.92)	Thought of the land being wasted (*M* = 4.79, *SD* = 8.03, *Mdn* = 0); exposure to air pollution (*M* = 4.88, *SD* = 6.96, *Mdn* = 0)
4	Anti-social behaviour (*M* = 29.96, *SD* = 19.49, *Mdn* = 24.5); traffic (*M* = 19.37, *SD* = 15.73, *Mdn* = 19, *Mdn* = 17.5)	Thought of the land being wasted (*M* = 5.54, *SD* = 11.52, *Mdn* = 0); conflict among users (*M* = 6.54, *SD* = 8.89, *Mdn* = 0)
5	Traffic (*M* = 27.05, *SD* = 7.73, *Mdn* = 27.78); anti-social behaviour (*M* = 26.15, *SD* = 17.73, *Mdn* = 26.67)	Thought of the land being wasted (*M* = 3.33, *SD* = 6.14, *Mdn* = 0); conflict among users (*M* = 3.89, *SD* = 5.14, *Mdn* = 0)
6	Anti-social behaviour (*M* = 23, *SD* = 22.50, *Mdn* = 20); traffic (*M* = 22.92, *SD* = 22.13, *Mdn* = 20)	Thought of the land being wasted (*M* = 5.17, *SD* = 9.02, *Mdn* = 0); conflict among users (*M* = 7.29, *SD* = 11.97, *Mdn* = 0)

A Kendall’s W test confirmed that the focus groups did not agree on how they assigned points to the different EDS, *W* = 0.336, *p* = 0.013. The complete list of reasons given by the participants in assigning points to specific ES and EDS in all the valuation exercises can be found in the Supplementary materials.

#### Group to individual

When the participants were asked to distribute the points to the park ES as a group, considering each other’s opinions, they assigned higher points to ecotourism, enjoyment and spending free time, sports and physical fitness, and relaxation and mental recreation. In contrast, they assigned lower points to the use of the park as a parking space and its ability to improve the residents’ non-economic quality of life, provide information for cognitive development, and stimulate residents’ interest in history and culture (Table 3). Based on a Kendall’s W test, the focus groups did not agree on how they assigned points to the different ES, *W* = 0.505, *p* < 0.001.

In terms of the EDS, as groups, they assigned higher points to anti-social behaviour and the expensive maintenance of the park, and lower points to the thought of the land being wasted with the construction of the park and exposure to air pollution (Table 4). However, based on a Kendall’s W test, the focus groups did not agree on how they assigned points to the different EDS, *W* = 0.546, *p* < 0.001.

#### Individual to group

When the participants were asked to distribute the points to the park ES again individually, they assigned higher points to those related to ecotourism, sports and physical fitness, relaxation and mental recreation, and enjoyment and spending free time. In contrast, they assigned lower points to ES related to the use of the park as a parking space, its ability to improve the non-economic quality of life of the residents, revenue for the city, and information for cognitive development (Table 3). According to a Kendall’s *W* test, the focus groups did not agree on how they assigned points to the different ES, *W* = 0.588, *p* < 0.001.

In terms of the EDS, participants were generally more worried about traffic and anti-social behaviour and less worried about the thought of the land being wasted because of the park’s construction and exposure to air pollution (Table 4). Similar to ES, the focus groups did not agree on how they assigned points to the different EDS, according to a Kendall’s W test, *W* = 0.393, *p* = 0.004.

#### Individual to future generations

Table 3 lists the park ES that the participants valued the most and the least when considering future generations. A Kendall’s W test determined that the focus groups did not agree on how they assigned points to the different ES, *W* = 0.470, *p* < 0.001. Some participants said that they assigned higher points to sports and physical fitness because they predicted that future generations would value fitness. They also assigned higher points to enjoyment and relaxation because they believe that future generations will be more prone to stress. Participants also highlighted that the park’s ability to promote local identity and cultural heritage, stimulate residents’ interest in history and culture, serve as a place for city events, provide information for cognitive development, increase the green areas in the city, and provide revenue for locals are also important. Some participants expect that the completion of the coliseum (shaped like a pot or “*banga”* in Filipino, where the city got its name) would lead to the promotion of the city’s local identity and stimulate the locals’ interest in the city’s history and culture. Some of them also believe that there is a need for more research about nature and parks and that the future needs more greens because of climate change:

Studies and research about the environment; because as the world becomes more modernised, we lose our trees [greens].

I gave more points to the addition of greens because I think in the future, we need to value greens like trees more because of climate change.

Table 4 lists the park EDS that the participants were most concerned and least worried for future generations. A Kendall’s W test confirmed that the focus groups did not agree on how they assigned points to the different ES, *W* = 0.351, *p* = 0.010. Participants expressed that they imagine gangs and youth staying late in the park at night will still be present in the future; thus, they still worry about anti-social activities. They also predict that the park will get more popular with the coliseum’s completion, attracting more people and vehicles and causing traffic. They were less worried about the conflict among users as they anticipate that a booking system will have been created in the future. Some participants asserted that they increased points for incomplete facilities since they are not sure if the park’s facilities will be able to accommodate the expected increase in visitors.

#### Group to future generations

Overall, when the participants were asked to distribute the points to the park ES as a group, thinking about the future generations and considering each other’s opinions, they assigned higher points to those related to sports and physical fitness, ecotourism, revenue for locals, and enjoyment and spending free time. In contrast, they assigned lower points to ES related to the use of the park as a parking space and the park’s capacity to improve the non-economic quality of life of the residents, bring revenue for the city, and provide aesthetic information (Table 3). The focus groups did not agree on how they assigned points to the different ES based on a Kendall’s W test, *W* = 0.421, *p* < 0.001.

In terms of the EDS, participants were generally more worried about traffic and anti-social behaviour and less concerned about the thought of the land being wasted with the construction of the park and the conflict among users (Table 4). The focus groups also did not agree on how they assigned points to the different EDS, based on a Kendall’s W test, *W* = 0.458, *p* = 0.001.

#### Individual to future generations (after discussions)

Overall, when the participants were asked to distribute the points to the park ES again after their valuation as a group and considering future generations, they assigned higher points to those related to ecotourism, sports and physical fitness, enjoyment and spending free time, and relaxation and mental recreation. In contrast, they assigned lower points to ES related to the use of the park as a parking space and the park’s ability to improve the non-economic quality of life of the residents, provide revenue for the city, and provide space for events (Table 3). The focus groups did not agree on how they assigned points to the different ES based on a Kendall’s W test, *W* = 0.463, *p* < 0.001.

In terms of the EDS, participants were generally more worried about anti-social behaviour and traffic and less concerned about the thought of the land being wasted with the construction of the park and conflict among users (Table 4). The focus groups also did not agree on how they assigned points to the different EDS, based on a Kendall’s W test, *W* = 0.267, *p* = 0.046.

#### Comparison of valuation exercises

##### Value source and constituency

Boxplots comparing the points assigned by participants to the different park ES and EDS in the six valuation exercises are presented in the Supplementary materials. Friedman tests (Friedman 1937) were run to determine if there were differences in how participants assigned points to each ES and EDS in the first five valuation events, where the combinations of valuation source and constituency were modified. For t.

he ES, it was found that there were significant differences in the points assigned by the participants to ES1 (ecotourism) [χ^2^(2) = 12.455, *p* = 0.014], ES5 (aesthetic information) [χ^2^(2) = 15.038, *p* = 0.005], ES6 (information for cognitive development) [χ^2^(2) = 14.836, *p* < 0.005], ES11 (revenue for locals) [χ^2^(2) = 21.703, *p* < 0.001], ES13 (parking space) [χ^2^(2) = 21.4, *p* < 0.001], and ES15 (increasing green areas) [χ^2^(2) = 13.141, *p* = 0.011] across the five valuation exercises. Pairwise comparisons were performed with a Bonferroni correction for multiple comparisons. Post hoc analysis revealed statistically significant differences on the points that they assigned to ES5 (aesthetic information) in the fifth (group to future generations) (*M*d*n* = 0) and first (individual to self) (*M*d*n* = 5.50) valuation exercise (*p* = 0.022). There were also statistically significant differences on the points that they assigned to ES11 (revenue for locals) in the first (individual to self) (*M*d*n* = 0) and fifth (group to future generations) (*M*d*n* = 9.09) (*p* = 0.002), fourth (individual to future generations) (*M*d*n* = 0) and fifth (group to future generations) (*M*d*n* = 9.09) (*p* = 0.007), and second (group to individual) (*M*d*n* = 2.78) and fifth (group to future generations) (*M*d*n* = 9.09) valuation exercise (*p* = 0.014).

For EDS, it was found that there were significant differences in the points assigned to EDS1 (expensive maintenance) [χ^2^(4) = 18.248, *p* = 0.001], EDS2 (traffic) [χ^2^(4) = 14.688, *p* = 0.005], EDS5 (thought of the land being wasted) [χ^2^(4) = 12.558, *p* = 0.014], and EDS6 (exposure to pollution) [χ^2^(4) = 16.223, *p* = 0.003] across the five valuation exercises. Pairwise comparisons were performed with a Bonferroni correction for multiple comparisons. Post hoc analysis revealed statistically significant differences on the values that participants assigned to EDS1 (expensive maintenance) in the fourth (individual to future generations) (*M*d*n* = 8) and second (group to individual) (*M*d*n* = 22.73) (*p* = 0.003) and in the fifth (group to future generations) (*M*d*n* = 10.56) and second (group to individual) (*M*d*n* = 22.73) valuation exercise (*p* = 0.019). There were also statistically significant differences on the values that they assigned to EDS2 (traffic) in the first (individual to self) (*M*d*n* = 12) and fifth (group to future generations) (*M*d*n* = 27.78) valuation exercises (*p* = 0.005) and to EDS6 (exposure to pollution) in the third (individual to group) (*M*d*n* = 3.57) and fifth (group to future generations) (*M*d*n* = 14.59) valuation exercises (*p* = 0.026). There were no statistically significant differences on the points that participants assigned to EDS5 (thought of the land being wasted).

## Discussions

Wilcoxon signed-rank tests (Wilcoxon 1945) were performed to determine if there are differences in the points assigned by the participants to the different park ES and EDS before and after deliberating with other participants. Data from the fourth and sixth valuation exercises were used for this analysis since both have the individual as the source and future generations as the constituency of the valuation. It was found that there were no significant differences between the points that the participants assigned to the different park ES and EDS before and after discussions with other participants when considering the future generations (see Supplementary materials).

## Future generations

Wilcoxon signed-rank tests were conducted to determine if there are differences in the points assigned by the participants to the different park ES and EDS when they were asked to consider the other participants in the focus group (third valuation) and when they were asked to consider the future generations (fourth valuation). For the ES, there was a statistically significant median decrease in the points that the participants assigned to ES1 (ecotourism) (*z* = -2.588, *p* = 0.010) and a statistically significant median increase in the points assigned to ES15 (increase in green areas) (*z* = 2.165, *p* = 0.030) when they were asked to consider the welfare of the future generations. For the EDS, there was a statistically significant median increase in the points that the participants assigned to EDS2 (traffic) (*z* = 2.232, *p* = 0.026) and EDS6 (exposure to air pollution) (*z* = 2.666, *p* = 0.008) when they were asked to consider the welfare of the future generations.

Considering only the mean points for each park ES, it can be generalised that the participants consistently assigned high values to four ES and low values to two ES. The high-valued ES were ecotourism, enjoyment and spending free time, sports and physical fitness, and relaxation and mental recreation, while the low-valued ones were the use of the park as a parking space and the park’s capacity to improve the residents’ non-economic quality of life. For EDS, participants consistently assigned high values to expensive maintenance and anti-social behaviour in the first and second valuation exercises and traffic and anti-social behaviour from the third to the sixth valuation exercise. The thought of land being wasted because of the park’s construction was valued consistently low across the six valuation exercises. However, the Kendall’ W test results reveal that the focus groups overall did not agree on how they distributed points to the different ES and EDS in each valuation exercise. These findings suggest that each focus group is unique in assigning values to the park’s ES and EDS.

The analyses confirm significant differences in the values assigned to several ES and EDS across the first five valuation exercises where the source and constituency of the valuation were shifted. These findings support Brown’s (1984) premise that assigned values depend on the source and the constituency of valuation and that *“even though one has a natural tendency to fall somewhere along the self-society continuum (i.e., thinking only of self-interests)…, the natural position along the continuum is altered by whomever the context of the valuation calls for the individual to represent”.* These results then highlight the need to specify the source and constituency when conducting socio-cultural valuation and reporting and comparing assigned values to ES and EDS. This study is the first to test the different valuation source and constituency combinations suggested by Brown (1984), consequently, there are no studies to compare the results to. However, a study by Schmidt et al. (2017), which investigated the relationship between ES values and land use preferences, concluded that other-oriented valuation (not specified if current or future generation) could result in higher assigned values to ES. In another study, Oteros-Rozas et al. (2014) suggested that other-oriented valuation leads to higher assigned values to ES related to social well-being rather than personal gains.

The valuation exercises with varying source and constituency proved useful in revealing the participants’ shared assigned values. According to Irvine et al. (2016), shared values represent the significance given to ecosystems beyond individual utility, but they may also appear indistinguishable from the self-interests of some individuals. This study adds that shared values transcend changes in value source and constituency. In the case of Jose Rizal Plaza, shared values are represented by the ES and EDS that were valued consistently high or low (*i.e.,* did not have significant differences) across the five valuation exercises. Those that were valued inconsistently could represent the ES and EDS that are unclear to the participants or the ones that could potentially cause stakeholder disagreements or active opposition from certain groups. The participants share a high appreciation for enjoyment and spending free time, sports and physical fitness, relaxation and mental recreation, social relationships, and local identity and cultural heritage. On the opposite, they share a low appreciation for stimulating interest in history and culture, revenue for the city, space for events, and improving the residents’ non-economic quality of life. Anti-social behaviour is the only shared high-valued EDS, while low-valued ones were conflict among users and incomplete facilities. The participants have varying opinions about ecotourism, aesthetic information, information for cognitive development, revenue for locals, and increasing green areas. These are expensive maintenance, traffic, the thought of the land being wasted, and exposure to pollution for the EDS. It can be noted that the shared ES and EDS values differ considerably from the ones observed only through the examination of aggregated values (*i.e.,* means). Some ES and EDS (e.g., ecotourism, expensive maintenance of the park) may even be misinterpreted as a shared value. These findings illustrate the point raised by Irvine et al. (2016) and Kenter et al. (2015) that shared values cannot be determined simply by aggregating individual values.

Results of the study show that there are no differences in the values assigned by participants to the park ES and EDS before and after discussions. Specifically, this is when the value source and constituency are kept the same. Discussions or deliberations have been shown to influence the assigned values to ES or EDS and even to policy options laid out to stakeholders (Bullock et al. 2018; Kenter et al. 2016a; Murphy et al. 2017; Raymond et al. 2014). The reason is that these activities allow for interaction, reasoning, and negotiations among stakeholders (Kenter et al. 2016b; Mavrommati et al. 2017). However, what is rarely mentioned explicitly in deliberative valuation studies is that discussions cause a shift in the value source and constituency, often from individual to self to group to individual or group to group. This shift then triggers a change in the set of held values that the stakeholders tap into when making preference decisions.

Results indicate a change in how the participants valued several ES and EDS when asked to consider future generations. These findings suggest that the “group” constituency could further be classified into those already known to the participants and those in the future. This opens the opportunity of incorporating intergenerational equity into the socio-cultural valuation of ES and EDS, and therefore into policy considerations. This opportunity is valuable as there is no way to elicit future generations’ preferences, and thus, they are consistently misrepresented in valuation studies (Mavrommati et al. 2017; O’Neill 2001).

While it generated many useful findings, it is essential to note that the study had a limited number of focus groups and participants. It is possible that not all stakeholder groups were represented well because the focus groups were promoted and administered online. There can also be a limitation on the level of participant interaction online. Moreover, since the focus groups were only conducted once, the study does not answer whether deliberation-influenced group values persist and eventually become shared values. Future studies should still aim to represent as many stakeholder groups as possible in the focus groups and conduct longitudinal surveys to acquire insights on the persistence of deliberation-influenced group values. Other aspects of the valuation context could also be experimentally modified to see how they influence value dynamics.

### Debriefing

When the participants were asked how they think the discussions affected their decisions in distributing points to the park ES and EDS, all of those who commented stated that other participants’ opinions influenced their decision-making. The complete list of translated excerpts from the focus groups related to the impact of discussions on valuing the park ES and EDS is in the Supplementary materials.

When asked what influenced their decisions in assigning points to the different park ES and EDS for future generations, the participants indicated that they thought about how these would benefit or disbenefit their children, grandchildren, the youth of the city, and all residents of the city. Some expressed their worries about the maintenance of the park:

I did not think about the people. I was more concerned with the maintenance, especially of the greens in the park... I wondered, ‘how can they [city office] be able to maintain them [the greens]’... I wish to see more greens in the park in the future.

I focused more on the EDS since I worry, ‘what if the government funds get exhausted in trying to resolve the EDS?’.

The participants were asked about their opinions on whether the park should be opened or closed during the pandemic. Few of them answered that it should be closed to lessen people’s movement and prevent the spread of the virus. Most of the participants think that it should be opened for the residents’ physical and mental health. They said that health protocols could be implemented to make sure that people are safe in the park. Some even suggested that a part of the park be converted into a COVID-19 isolation facility to maximise the use of its space (see Supplementary materials).

The general themes that emerged when the participants were asked what they have learned from the focus groups were the following: ES and EDS, appreciation of the park and the topic of the research, importance of discussions, the value of participation in decision-making, and future generations. Many of the participants mentioned that they had realised the unique benefits of the park to different kinds of people in the city because of the focus group. They also expressed their surprise to hear the benefits of the park that they never noticed before. They think that they now have a better understanding and awareness of the park’s benefits and disbenefits. Participants also cited that the focus groups led to a new-found appreciation of the park and the motivation to visit it more when the pandemic is over. They also commended the research topic, saying that it tackles a relevant issue, especially now that the city is becoming more commercialised. Few participants mentioned that they developed a more comprehensive perspective on the park’s value after the discussions. One participant pointed out the importance of research and participation in the city’s decision-making (for the park’s design). Participants also stated that the focus group sparked concern for future generations and prompted them to think about their welfare:

This focus group stresses the importance of research [in decision-making]. There needs to be planning in decision-making… If there’s no planning, there will be negative consequences or there will be some points that will be missed [not considered].

This focus group enabled me to consider the future generations or the future of the next generations. ‘How will future generations know Calamba City? Is it going to be historical landmarks?’ This focus group is an eye-opener for me..

Participants noted that other participants’ opinions somehow influenced their preferences and that they learned lessons from the focus groups. These findings support the claims (see Irvine et al. 2016; Kenter et al. 2015) that deliberations lead to social learning and the formation of shared values (deliberation-influenced shared values). It also validates that socio-cultural valuation studies can also aid in information dissemination and awareness-raising (Walz et al. 2019). Participants also expressed that the focus groups stimulated their concerns about the welfare of future generations. Mavrommati et al. (2020) also assert that deliberative approaches effectively integrate future considerations into the current environmental choices. It is also clear from the results that the park’s closure due to the pandemic has influenced the participants’ valuation. Most of the shared high-valued ES were the same ones that they reasoned why the park needs to be opened—enjoyment and spending free time, sports and physical fitness, relaxation and mental recreation, and social relationships.

## Conclusion

This study examined how deliberative socio-cultural valuation can elicit the value that stakeholders assign to a park’s ES and EDS. In addition, it illustrated how these assigned values could change in different situations. Although the study focused on a single case study in the Philippines, its findings are of general value to any city establishing and maintaining urban parks. Based on the study’s findings, deliberative socio-cultural valuation with varying sources and constituencies effectively reveals shared assigned values to ES and EDS. In the case of Jose Rizal Plaza, stakeholders share a high appreciation of the following ES: enjoyment, sports and physical fitness, relaxation and mental recreation, social relationships, and local identity and cultural heritage. For EDS, they share a serious concern with anti-social behaviour. The study also found that discussions alone do not impact assigned values, but it is the change in the source and constituency of the valuation that causes a shift. There is, therefore, a need for future studies to exercise caution in eliciting, reporting, and comparing values. This study argues that deliberative socio-cultural valuation is a valuable tool in assessing shared assigned values to ES and EDS and will help make more inclusive and well-informed decisions about managing natural and human-made ecosystems.
